# Therapeutic Properties of Stingless Bee Honey in Comparison with European Bee Honey

**DOI:** 10.1155/2018/6179596

**Published:** 2018-12-26

**Authors:** Fatin Aina Zulkhairi Amin, Suriana Sabri, Salma Malihah Mohammad, Maznah Ismail, Kim Wei Chan, Norsharina Ismail, Mohd Esa Norhaizan, Norhasnida Zawawi

**Affiliations:** ^1^Laboratory of Molecular Biomedicine, Institute of Bioscience, Universiti Putra Malaysia, 43400 Serdang, Selangor, Malaysia; ^2^Enzyme and Microbial Technology Research Center, Faculty of Biotechnology and Biomolecular Sciences, Universiti Putra Malaysia, 43400 Serdang, Selangor, Malaysia; ^3^Department of Microbiology, Faculty of Biotechnology and Biomolecular Sciences, Universiti Putra Malaysia, 43400 Serdang, Selangor, Malaysia; ^4^Department of Food Science, Faculty of Food Science and Technology, Universiti Putra Malaysia, 43400 Serdang, Selangor, Malaysia; ^5^Department of Nutrition and Dietitics, Faculty of Medicine and Health Sciences, Universiti Putra Malaysia, 43400 Serdang, Selangor, Malaysia

## Abstract

Both honeybees (*Apis* spp.) and stingless bees (*Trigona* spp.) produce honeys with high nutritional and therapeutics value. Until recently, the information regarding potential health benefits of stingless bee honey (SBH) in medical databases is still scarce as compared to the common European bee honey (EBH) which is well known for their properties as therapeutic agents. Although there have been very few reports on SBH, empirically these products would have similar therapeutic quality as the EBH. In addition, due to the structure of the nest, few studies reported that the antimicrobial activity of SBH is a little bit stronger than EBH. Therefore, the composition of both the types of honey as well as the traditional uses and clinical applications were compared. The results of various studies on EBH and SBH from tissue culture research to randomised control clinical trials were collated in this review. Interestingly, there are many therapeutic properties that are unique to SBH. Therefore, SBH has a great potential to be developed for modern medicinal uses.

## 1. Introduction

**Figure 1:**
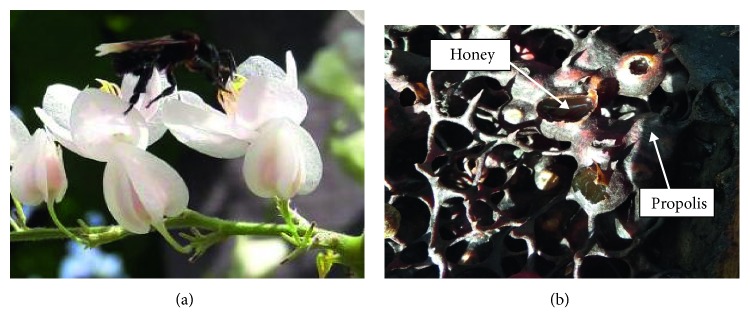
(a) Stingless bee. (b) Stingless bee honey in the nest.

Honey is an important natural food product since ancient times and is known for its nutritional and therapeutic values. It is produced from liquid plant exudates which is gathered, modified, and stored by different types of bees [1]. Among all, European honeybees (*Apis mellifera*) and stingless bees (Figure 1) are the two most common bees. The European honey bee is a member of a group of bees in the genus of *Apis* whereas stingless bee can be classified into two genera, namely, the *Melipona* and the *Trigona* [2]. Both have important role in flower pollination [3–5]. Honey produced by stingless bees is known with various names such as Meliponine honey, stingless bee honey (SBH) pot-honey, and also *Kelulut* honey (in Malaysia). It is one of the valuable bee products and is attributed with some medicinal properties by ancient peoples [6]. Since the 20th century, various studies on the chemical and biological properties of honey had been conducted due to their extraordinary antibacterial, bacteriostatic, anti-inflammatory, wound, and sunburn healing effects [7].

In the modern time, due to its outstanding medicinal value, honey has been both exported and imported globally. Like other food supplements, the therapeutic effects of honey also depend on its quality [8]. Sensorial, chemical, physical, and microbiological characteristics are generally used to determine the quality of honey. Even within same species, the quality parameters of honey produced varies and dependent on other factors such as the maturity achieved in the bee nest or hive during the harvesting season, climatic and geographic factors, and other elements that affects the floral abundance [6]. For example, in the United States only more than 300 different types of honey can be found, each with unique flavour and appearance, depending to its floral sources. Currently, researchers have established that the main reason behind the diverse color, flavour, and also functional properties of honey is mainly due to its phenolic composition rather than variation in other components, such as carbohydrates and proteins [9].

Most well-researched natural honeys are the European bee honey (EBH) such as Manuka honey, jelly bush honey, African jungle honey, and Malaysian Tualang honey. As compared to the benefits of EBH (Manuka honey) which has been internationally recognised [10], the potential health benefits of stingless bee honey (SBH) are only recently gaining a lot of attention [11–13]. Despite that, a variety of phenolic compounds such as gallic acid, caffeic acid, catechin, and apigenin have also been reported in both honeys [14, 15]. Honey exhibits significant antioxidant, anticancer, and antiatherogenic activities which may be attributed partly to these compounds [16–18]. For example, SBH has been proven to show peculiar antioxidant activities and exhibits outstanding ability in reducing inflammation and infection [19]. Therefore, in this, we describe the health effects of stingless bee honey in comparison with honey produced by the European honey bee. The importance of polyphenols in honey as well as their potential mechanisms in treating certain diseases is also discussed.

## 2. Physicochemical Properties of Stingless Bee Honey and European Bee Honey

Alongside water and sugars as the major contents, both SBH and EBH are also known to be rich in vitamins, enzymes, amino acids, and minerals, with almost 200 different compounds were reported in both honeys. Usually, honeys are acidic due to its low pH (∼pH 4), and are made up of 80% sugars and 17% water, while the remaining 3% is contributed by various enzymes, acids, and minerals [20]. However, the composition of honey differs according to the floral source and origin [21]. For example, in Thailand [22], it was reported that the composition and quality of Thai SBH differs from EBH.

Fructose is reported as the most abundant sugars found in both honeys with approximately 31–39% of various sugars in honey [23]. Other than fructose and glucose, many studies had reported the presence of various disaccharides and oligosaccharides in honey. Inulobiose, kestose, and nystose are some of the fructooligosaccharides identified in Malaysian Tualang honey (*Apis dorsata*) while New Zealand honey contains isomaltose and melezitose [7, 24] as well as raffinose has been found in Italian honey [25]. Minerals or trace elements present in honey are potassium, zinc, phosphorus, calcium, sodium, magnesium, sulphur, copper, iron, and manganese [26].

Because of its unique flavour and high nutritional value, the price of honey is relatively higher than other sweeteners. Adulteration of honey is a serious problem which currently has a significant impact on economy as well as irrefutable nutritional and organoleptic ramification. Lack of knowledge regarding composition and physicochemical characteristics of SBH worldwide has led to its adulteration and falsification [27]. Full data or detail information on the physicochemical properties of honey is important to decrease the possibility of adulteration. The data obtained from various studies are being used to develop new regulatory standards of SBH [27]. The different physicochemical characteristics of EBH, namely, Tualang honey and Manuka honey and SBH are summarized in Table 1.

## 3. General Nutritional Properties and Dietary Values of Honey

The high nutritional and therapeutic value of honey has been well documented from long time ago. Before cane sugar is being used widely, honey has always been the choice of natural sweetener. Honey is a concentrated solution of reducing sugars such as fructose and glucose and nonreducing sugars such as sucrose and maltose. Among all, fructose and glucose represent the largest proportion of honey composition. As the results, honey tastes sweeter than sucrose as sweetening power of fructose is 1.3 while sugar cane-derived sucrose is only 1 [33], making it a better substitute as sweetener, with higher nutritional value as compared to commercial sugar. Despite its reported high fructose level, 48.1% of SBH contained lower levels of reducing sugar when compared to *Apis mellifera* honey (EBH) standards [8, 34].

Honey is one of the outstanding sources of energy due to its high sugar concentration. The energy input represented by honey is approximately 300 kcal per 100 g [35]. The high calorific value of honey makes it suitable for athletes as it contains readily absorbed glucose which will be converted into energy in a short time [33]. Other than it is used as a source of energy, honey is also important for bones and teeth. It helps in absorption of calcium and magnesium retention which may contribute to stronger bone and better dental calcification. This is due to the presence of nondigestable carbohydrate such as raffinose that produces short-chain fatty acid (SCFA) as the by-product from the fermentation process in the caecum and colon. The SCFA helps to lower the intestinal pH and creating a favourable environment that increases mineral such as calcium solubility and absorption [36].

## 4. Polyphenols of Stingless Bee Honey and European Bee Honey

Flavonoids and phenolic acids are the most common group of polyphenols that are previously detected in both honeys. As for the flavonoid groups, only the flavonols (such as myricetin, kaempferol, 8-methoxy kaempferol, quercetin, isorhamnetin, quercetin-3-methyl ether, quercetin-3, 7-dimethyl ether, pinobanksin, rutin, and galangin), flavones (such as genkwanin, luteolin, apigenin, tricetin, and chrysin), and flavanones (such as pinocembrin and pinostrobin) were previously detected in honey. Meanwhile, among the phenolic acid group, the hydroxybenzoic acids such as methyl syringate, gallic acid, syringic acid, benzoic acid, and 4-hydroxybenzoic acid and hydroxyl-cinnamic acids such as chlorogenic, vanillic, caffeic, p-coumaric, and ferulic acids are present in various honey samples [37]. Common polyphenols detected in both honeys, each with different potential therapeutic effects, are summarized in Table 2.

Honey is known for its antioxidant activity. A prior investigation indicated that the total antioxidant activity of honey is primarily provided by its phenolic composition, rather than vitamin C and other components [12]. The antioxidants that occur naturally in honey are flavonoids, phenolic acids, enzymes (e.g., glucose oxidase and catalase), ascorbic acid, carotenoid-like substances, organic acids, Maillard reaction products, amino acids, and proteins [40, 50] Several *in vivo* studies strongly suggested that long-term consumption of diets rich in these types of polyphenols significantly ameliorates the adverse effects of several liver-, heart-, kidney-, brain-, and pancreas-associated diseases as well as those of genetic disorders such as tumors and cancer [38, 51].

## 5. Traditional Uses of Stingless Bee Honey and European Bee Honey

Natural honey has been used to prevent and treat variety of ailments since years ago [52]. For example, newborn babies were fed with EBH as a supplement [53], meanwhile EBH also has been used by Ayurvedic physicians as alternatives for medicines, and it was recommended to satisfy the immediate calorie demand for the patients [52].

A study by Reyes-González et al. [54] reported that the SBH is also known for its medicinal value and uses in food. According to the natives, after being extracted, the honey is often consumed along with a hot drink, or even alone. As medication, SBH is employed for treating various sicknesses by combining this honey with different ingredients such as lemon, agave mezcal, and pulp of *Crescentia alata*. The combination was used to treat cold, cough, and respiratory illness such as bronchitis. Besides that, the SBH is extensively used as a fundamental part of medicine by the Maya traditional doctors as remedy for high fever, treatment for wounds and burns, and also the cure for poisonous stings [55]. Despite being known as functional food, honey is also credited with many therapeutic values.

## 6. Therapeutic Effects of Stingless Bee Honey and European Bee Honey and Their Polyphenols

Microbial resistance towards modern antimicrobial drugs is rising and had become the topic of interest among the scientists in which scientists are developing novel drugs with less or no microbial resistance, and also have broad-spectrum inhibition activity. Despite the traditional uses of honey as therapeutic agents, honey is recently acknowledged in modern medicine development [56] due to its valuable nutritional quality. It also portrays potential properties against reactive oxygen species (ROS), acts effectively as anti-inflammatory and antibacterial agents against bacteria and fungi and a potential substitute in reducing coughs and wound curing [56]. The common therapeutic properties of most honeys are more likely based on their floral origins. Since few years back, the role of honey in wound healing has been widely studied and proven to be the most effective therapeutic effects of honey [57]. Previously, honey has been used to treat wound infection and promotes wound healing by the Russians during World War 1. Mixture of honey and cod liver oil has shown to be effective by the Germans, in treating ulcers and burns [58]. In addition, honey is world widely known for its roles in the treating of famous ophthalmological diseases such as keratitis, conjunctivitis, corneal injuries, blepharitis, and chemical and thermal burns to eyes [59, 60].

Honey contains various polyphenols, which differs according to the origin and bee species [48]. Various polyphenols, of which some are also detected in honey, have been proven to curb the development of many diseases. They perform this action via several specific mechanisms such as regulation of a specific gene expression or altering metabolic pathways by means of promoting or blocking specific pathways [37]. However, differences in honey samples may affect the type of polyphenols found in honey. As one type of honey might not contain all of the polyphenols described and the protective effects of polyphenols are varied, it is advisable to consume variety of honey samples. The therapeutics effects of SBH and EBH such as antidiabetic, wound healing, anticancer, treatment of eye disease, and effects of fertility as proven by many scientific studies will be described as below. The therapeutics effects of both honeys are summarized in Table 3.

### 6.1. Antidiabetic

Hyperglycemia, deranged lipid profiles, and inadequate insulin production by the pancreas are the characteristics of chronic metabolic disorders, diabetes mellitus [60]. Numerous studies have reported the antidiabetic effects of honeys particularly from EBH. EBH from Nigeria, for example, had shown increment in high-density lipoprotein (HDL) cholesterol level, while hyperglycemia, triglycerides (TGs), very low-density lipoprotein (VLDL) cholesterol, non-HDL cholesterol levels, coronary risk index (CRI), and cardiovascular risk index (CVRI) were reduced in alloxan-induced male diabetic Wistar rats [61]. Pretreatment of EBH known as Gelam honey produced by *Apis dorsata* on pancreatic hamster cells has been reported to modify the inflammation-induced insulin signalling pathways [62]. Promising antihyperglycemic effects of EBH in the diabetic rabbit model has been reported as blood glucose levels, and other related parameters were significantly reduced in this study [63]. Apart from its wound healing effects, the EBH known as Tualang honey has great antioxidant activities towards the organs of chemically induced diabetic rats such as pancreas. The hypoglycemic effect of Tualang honey in diabetic animal model might also be contributed by the protective effects against oxidative stress of the pancreas [89].

*α*-Amylase and *α*-glucosidase are the two main enzymes that are involved in elevation of blood glucose. The inhibition of these two enzymes indicates a good antidiabetic effect as it helps to reduce the levels of blood glucose. In a comparative study, the antidiabetic properties of EBH and SBH were analysed using *in vitro α*-amylase and *α*-glucosidase enzyme inhibition assays, whereby SBH was found to exhibit the highest inhibition of both enzymes, indicating a better antidiabetic properties as compared to other EBH honeys used in this study [64]. The complex starch molecules will be converted into simple sugars by these enzymes; therefore, a competitive inhibition between *α*-amylase and *α*-glucosidase with the phytochemicals in the honey could prevent the rise of blood sugar level at a faster rate [64]. SBH also showed remarkable antidiabetic effects *in vivo* as reported by Aziz et al. [65], where administration of this honey to diabetic male rats did not increase the level of fasting blood glucose, total cholesterols, triglyceride, and low-density lipoprotein.

Honey and other medicinal plants which are reported to contain many bioactive compounds [90] were used traditionally and are still being used as alternative to treat diabetes [91]. Evidences from scientific studies showed that dietary polyphenols are useful in treatment of diabetes mellitus. Out of many polyphenols found in both honeys, only few of them, such as quercetin, apigenin, luteolin, catechin, rutin, and kaempferol, are detected to exhibit antidiabetic properties. This is achieved via several mechanisms to reduce blood glucose levels [37]. These include several important mechanisms such as *α*-amylase and *α*-glucosidase and gluconeogenic enzymes inhibition [92, 93], enhancement of pancreatic b-cell protection and glucose uptake [94, 95], and reduction of oxidative stress [95].

The potential role of honey polyphenols in inhibiting *α*-amylase and *α*-glucosidase enzymes that facilitate carbohydrate breakdown has been confirmed. Quercetin successfully inhibits the *α*-glucosidase enzymes and reduces maltose-induced postprandial hyperglycemia in patients diagnosed with type 2 diabetes mellitus (T2DM) [96]. Meanwhile, another study proved the inhibition of *α*-amylase and *α*-glucosidase by luteolin and luteolin-7-0-glycoside [97]. The level of blood glucose in an animal's body is controlled by pancreatic *β*-cells, so any changes in this process will lead to diabetes mellitus. Therefore, to control diabetes mellitus, pancreatic *β*-cells must be protected. Honey polyphenols such as quercetin also helps to protect pancreatic *β*-cells in numerous studies. For example, quercetin administration at a dose of 10–15 mg/kg for ten days in streptozotocin-induced rats resulted in increment of pancreatic *β*-cell numbers [98].

### 6.2. Wound Healing

Honey therapy has been used to treat septic wounds, surgical wound, or wounds of abdominal wall and perineum due to its excellent wound healing properties. Previously, it is also being used in treating abrasion, amputation, and burns [58, 73, 99]. In general, the oedema, inflammation, and exudation that commonly occur in all types of wounds were reduced by honey in order to improve the wound healing effects. The growth of epithelial cells and fibroblasts was also stimulated by honey [67, 100].

In Iran, the EBH was topically applied on wounds created on rabbits. As the results, the oedema and necrosis seems to lessen, and infiltrations of polymorphonuclear and mononuclear cell become fewer. The wound contraction also seems to improve, with better epithelialisation, and lower concentrations of glycosaminoglycan and proteoglycan [67]. Moreover, application of the EBH on wounds made on the animal model showed faster healing activity as compared to nitrofurazone or sterilized petrolatum [101].

In another study, EBH, ampicillin ointment, and saline were used to treat full-thickness skin wounds created on buffalo calves. The healing efficacy is superior to EBH-treated wound in comparison with ampicillin and saline treatments where least inflammation, most rapid fibroblastic and angioblastic activity, and epithelialisation were observed [101]. Similarly, a study by Sarkar et al. [66] investigated the effect of EBH on collagen homeostasis restoration in diabetic animal whereby a full-thickness wound was created on streptozotocin-induced rat. Topical application of normal saline, EBH, and povidone iodine on wound was compared. The findings showed that the EBH might be predominantly helpful in synthesis, glycation, deposition regulation, and collagen quality alike normal skin. Honey application was also proven to accelerate diabetic healing process [66].

Moreover, a recent study had shown enhanced healing of electroscalpel-induced wound of Wistar rats by EBH treatment, as compared to silver sulfadiazine which is being used as positive control [68]. Another *in vivo* study also proved that oral administration of EBH to measure the healing of colonic anastomosis in rats, had shown increment of the tensile strength measured by bursting pressure, increased fibroblast count and lowered mean of inflammatory cells count in rats supplemented with honey after the surgical procedure in comparison with the control group [70, 102]. The increase in tensile strength in EBH-treated wound might be caused by the increase in collagen concentration, produced by fibroblasts.

A few *in vitro* studies revealed the substantial antimicrobial activity of SBH, which could also suggests the possible wound healing activity of this honey [71, 103, 104]. In addition, combination of SBH and other substances such as antibiotic ampicillin or garlic extract, rather than these substances alone, showed more effective effects in inhibiting the growth of *S. aureus*, which is the most common pathogenic bacteria causing wound infection [11, 71]. Honey is useful as wound dressing as it helps to stimulate the healing process and can clear the infection quickly for it portrays better cleansing activity. Besides, it has proven anti-inflammatory activity and plays a great role in stimulating tissue regeneration [58, 73, 99].

### 6.3. Anticancer

Honey, as described by many scientific evidences, may be considered as a great chemopreventive agent. Chemoprevention may be described as the usage of natural or synthetic compounds in order to decrease the risk of cancer development [104].

Scientific evidence has proven that superoxide anion radical and inflammation can cause somatic mutation which will eventually evolve to initiate cancer. Due to its excellent anti-inflammatory activity, the anticancer effect of honey was also being investigated. According to Ahmed and Othman [105], as honey is known with its apoptotic, antiproliferative, antioxidant, anti-inflammatory, estrogenic, and immunomodulatory activity, these might be considered as the possible mechanisms of how honey prevent the progress of the cancer formation.

In one experiment, the human hepatoma cell (HepG2) is treated with the EBH. The results showed that the viability of the cells is greatly reduced in a dose- and time-dependent manner. Hepatic injury may be initiated by oxidant molecule such as nitrogen oxide, through reactive oxygen species (ROS) and lipid peroxidation products, and these molecules may also cause inhibition of apoptosis by various pathways [74]. As expected, the level of radical nitrogen oxide in the culture supernatant was reduced by honey treatment. Therefore, it can be concluded that the anticancer effect of honey might be due to its antioxidant activity, which helps in curbing the initial formation of cancer. In another study, a rat model was induced with mammary cancer; however, oral administration of EBH was proven to prevent the mammary cancer induced with 7, 12-dimethylbenz[a]anthracene (DMBA). An 18-week laboratory test reveals that EBH consumption had significantly lowered the rate of incidence, the efficacy to multiply, and the tumor size in rats of DMBA-induced mammary cancer. In conclusion, the antioxidant activity of EBH might also be the reasons of the protective effect against DMBA-induced mammary cancer [15, 75].

Meanwhile, SBH, which is also known for its antioxidant activity, prevents the induction of colon cancer by azoxymethane (AOM) in rats. Aberrant crypt foci (ACF) act as a biomarker in identifying the colon cancer development. To observe the effect of SBH on ACF, SBH was administered orally (1183 mg/kg body weight) and had proven to reduce the total number of ACF and aberrant crypt and crypt multiplicity. Therefore, SBH is neither harmful nor toxic to the animal [76]. An *in vitro* study was also conducted to screen for the cytotoxic activity of different stingless bee products against five human cancer cell line, namely, BT474 (ductal carcinoma and lung undifferentiated cancer), HepG29 (liver hepatoblastoma), KatoIII (gastric carcinoma), and SW620 (adenocarcinoma), whereby the crude extracts of SBH showed great cytotoxicity effects towards HepG2 cell line, while propolis crude extracts exhibit high cytotoxicity effects towards all the human cancer cell line [77].

Polyphenols with anticancer effects that can be found in both honeys are quercetin, apigenin, chrysin, and luteolin [37]. The mechanisms that are involved in cancer prevention by these polyphenols include inhibition of cell proliferation [106], modulation of cancer signalling pathways [107], and induction of tumor cell apoptosis [108]. Uncontrolled cell proliferation had caused the cancer cells to increase at a faster rate; therefore, if the uncontrolled cell proliferation can be inhibited or reduced, cancer prevention is more likely to be successful. Polyphenols, which are also known for their antioxidant properties, are very helpful in preventing cell proliferation. For example, chrysin which is an important honey flavone helps to control the cell proliferation by activating p38-MAPK via accumulation of p21Wafi/Cip1 in C6 glioma cells of rats [37, 109]. Meanwhile, apigenin prevents proliferation of pancreatic cell as it helps to reduce levels of cyclin A, cyclin B, and the phosphorylated forms of cdc2 and cdc25, thereby arresting the G2/M phase of the cell cycle [37, 110].

### 6.4. Treatment for Ocular Diseases

A study demonstrated that the bacterial flora in the conjunctival sac of patients with cataract and scheduled for vitrectomy was successfully eradicated after continuous administration of 25% sterile honeydew honey (EBH) for 7 days [78]. Similarly, Albietz and Lenton [79] pointed to the fact that the EBH significantly reduced formation of the whole colony-forming units in the eyelids and conjunctivae in patients with dry eye syndrome after one and three months of therapy.

Recently, Bashkaran et al. [80] compared the anti-inflammatory and antioxidant effect of the EBH with a corticosteroid preparation (prednisone) in the treatment of alkali burn in rabbit eyes and confirmed the anti-inflammatory effects of this honey on experimental animals, with no significant difference between the two treatments. In a clinical study, in which 16 patients with oedema of the corneal epithelium who had not been indicated for a surgical procedure were subjected to local therapy with the EBH. The result indicated that all corneas manifested an immediate regression of the corneal oedema with EBH treatment [81]. Previously, a preliminary study was conducted by Vit [82], where SBH drop was applied on selenite-induced rats and had resulted in reducing the rate of the cataract progress, in 20% of the rats in the group that received honey for the opacification treatment. Despite that, SBH was also proven to reduce the infection time for eye diseases caused by *Staphylococcus aureus* and *Pseudomonas aeruginosa*, via *in vivo* studies using rabbit as the animal model [83].

Polyphenols inhibit the angiogenesis and inflammatory cytokines and also eye diseases by suppressing formation of reactive oxygen species (ROS) and upregulate antioxidative enzymes [111]. Polyphenols with anticataract properties are mainly flavonoids, phenolic acids, carotenoids, and vitamins [112, 113]. Quercetin and catechin are the specific polyphenols that portray promising effects against ocular diseases [84, 114]. Both quercetin and catechin are previously detected in SBH and EBH [14, 37] and could also be found in fruits and vegetables [84, 114]. Quercetin (3,3′,4′,5,7-pentahydroxyflavone) can inhibit hydrogen peroxide-induced cataracts while catechin derivatives inhibit cataracts in rats induced by *N*-methyl-*N*-nitrosourea [114].

### 6.5. Effects on Fertility

Honey has been shown to portray positive effects on fertility by means of enhancing the hormones related to fertility [84]. Sexual dysfunction and impaired fertility are among the adverse effects that have been associated with cigarette smoking, especially in males. A laboratory study reported that reproductive toxicity induced by cigarette smoke was alleviated by the oral consumption of EBH at 1.2 g/kg/day, which raised the successful intromission and ejaculation percentage in rats, thus resulting in increased fertility and mating rates [84]. Besides that, noise stress is one of the stress factors, which is known to hinder male reproductivity. Noise stress has negatively impacted the cells of testicular tissue by promoting the growth of apoptotic and necrotic cells. However, with EBH and vitamin E treatment, it was observed that the cells of mature male Wistar rats which have been exposed to noise stress are enhanced and found healthy. This suggests that EBH and vitamin E have good effects on the testis parenchyma as EBH and vitamin E reduced apoptosis and necrosis in cells affected by noise stress and thereby increased cell growth and activity [85].

In one study, the intake of Tualang honey (EBH) produced by *Apis dorsata* to restraint-stressed pregnant rats at 1.2 g/kg daily resulted in favourable condition on several parameters, especially in the level of corticosterone, outcome of pregnancy, and adrenal histomorphometry [86]. It is reported that alteration of gonadotropin levels in female rats was significantly restored with EBH administration at 1 g/kg. Regularly, diabetic rats suffer from low sperm quality; however, SBH administration to diabetic rats portrays improvement in sperm quality, with additional protective effects on spermatogenesis process even in diabetic condition. In nondiabetic rats, administration of SBH helps to increase the count of epididymal sperm count, the motility, and viability of the sperm. This could suggest for potential property of the fertility enhancer in the SBH. In conclusion, the SBH could be a great alternative in order to prevent sperm and testis damage in diabetic rats [88].

## 7. Conclusion

This current review of the SBH in comparison with EBH revealed a significant role of the SBH as a therapeutic agent in various health issues such as antidiabetic, wound healing, anticancer, treatment of eye diseases, and also in fertility. Studies have proven that the SBH has excellent potential and portrays beneficial effects as antimicrobioal, anticancer agent, improving hypertension, lipid profiles, and with some studies showing better antidiabetic effects than the EBH *in vivo*. In addition, other therapeutic properties are also at par or even significantly better from the much-researched EBH. In order to provide a major comprehensive understanding on the potential uses and benefits of the SBH, more systematic studies need to be carried out. Previously, studies on SBH were done using tissue cultures, animal models, and clinical trials to demonstrate the biotherapeutic activities. However, the information on its beneficial effects is still scarce. With regard to its benefits to human health, more scientific studies and clinical trials on human subjects need to be conducted to relay a better understanding in evaluating the potential of stingless bee honey as a therapeutic agent.

There are a plethora of areas to study for researchers who are interested in the biotherapeutic effects of the SBH. In terms of quality control, methods to authenticate pure SBH need to be developed. A rapid and destructive analysis technique is required to avoid possible adulteration by irresponsible manufacturers. In return, it is expected that a quality standard can be established by the identification of its bioactive component. Since SBH is rich in antioxidants, these substances might account for some of the potential health benefits portrayed by them. Therefore, innovative efforts should be taken to fully explore and utilize these benefits. Honey-based products should be diversified, such as making supplement capsules or tablets which contain probiotics isolated from the SBH that can aid in gastrointestinal health. These properties should also be made readily in the form of topical creams or gels for wound healing or other purposes.

## Figures and Tables

**Table 1:** Different physicochemical characteristics of European bee honey and stingless bee honey.

Physicochemical properties	European bee Honeys		Stingless bee honeys
	Tualang honey, *Apis dorsata* [28–30]	Manuka honey, *Apis mellifera* [31, 32]	*Trigona* spp. [6, 8, 27]
Appearance	Dark brown	Light dark brown	Amber brown
Moisture content (%)	23.30	18.70	25.00–31.00
pH	3.55–4.00	3.20–4.20	3.15–4.66
Total reducing sugars (%)	67.60	75.80	54.90–87.00
Glucose (%)	29.50	35.90	8.10–31.00
Fructose (%)	29.60	40.00	31.11–40.20
Sucrose (%)	0.60	2.80	0.31–1.26
Maltose (%)	7.85	1.20	ND
Calcium (%)	0.18	1.15	0.017
Potassium (%)	0.51	1.00	0.07
Sodium (%)	0.26	0.0008	0.012
Magnesium (%)	0.11	1.00	0.004
Specific gravity	1.34	1.39	ND
Electrical conductivity (mS/cm)	0.75–1.37	0.53	0.49–8.77
Hydroxymethylfurfural HMF (mg/kg)	46.17	400.00	8.80–69.00
Ash content (g/100 g)	0.19	0.03	0.01–0.12

ND: not detected.

**Table 2:** Common phenolic compounds with their potential health benefits found in both European bee honeys and stingless bee honeys.

Compound	Molecular formulae	Potential health benefits	References
Gallic acid	C_7_H_6_O_5_	Antioxidant	[38]
		Anti-inflammatory	
		Cardioprotective activity	
		Antimutagenic	
		Anticancer	

Caffeic acid	C_9_H_8_O_4_	Cardiovascular diseases treatment	[39]
		Anti-inflammatory effects	[40]
		Anticancer	[41, 42]
		Antidiabetic	[43]

Catechin	C_15_H_14_O_6_	Cardiovascular diseases treatment	[44]
		Antidiabetic potential	[45]
		Anti-inflammatory	

Apigenin	C_15_H_10_O_5_	Anti-inflammatory	[46]
		Antimutagenic	
		Treating cardiovascular diseases	[37]

Chrysin	C_15_H_10_O_4_	Improves cognitive deficits and brain damage	[47]
		Anticancer	

Cinnamic acid	C_9_H_8_O_2_	Improves cognitive deficits and brain damage effect	[47]
		Antimicrobial effect	[48]

Kaempferol	C_15_H_10_O_6_	Cardiovascular diseases treatment	[39]

*p*-Coumaric acid	C_9_H_8_O_3_	Anticancer activity	[41, 43]
		Improves cognitive deficits and brain damage effect	[47]

Quercetin-3-O-rutinoside (rutin)	C_27_H_30_O_16_	Antiallergic	[49]
		Anti-inflammatory	
		Antiproliferative	
		Antitumor	

**Table 3:** Summary of therapeutic properties of European bee honey and stingless bee honey from previous studies.

Properties	Honey types and bee species	Therapeutic effects	Reference
Antidiabetic	Nigerian honey (*Apis* spp.)	Increased high-density lipoprotein (HDL) cholesterol	[61]
		Reduced hyperglycemia, triglycerides (TGs), very low-density lipoprotein (VLDL) cholesterol, non-HDL cholesterol, coronary risk index (CRI), and cardiovascular risk index	
	Gelam honey (*Apis dorsata*)	Increased expression of phosphorylated JNK and JKK-*β*. Reduced expression of TNF‐*α*, IL‐6, IL‐1*β*, and Akt phosphorylation	[62]
		Expression of TNF-*α*, IL-6, IL-1*β*, and Akt phosphorylation	
	European bee honey (*Apis* spp.)	No effect on glucose level at low dosage	[63]
		Increased blood glucose at high dosage	
	European bee honey and stingless bee honey (*Apis cerana indica*, *Apis mellifera*, *Apis dorsata*, *Apis florae*, and *Trigona iridipennis*)	Higher percentage of inhibition against *α*-amylase and *α*-glucosidase enzyme (*Trigona sp*.)	[64]
	Stingless bee honey (*Geniotrigona thoracica*)	Prevent increased of fasting-blood glucose (FBG), total cholesterols (TC), TGs, and LDL levels	[65]
		Increased HDL and serum insulin levels	
		Decreasedchanges of histopathological and oxidative stress expression level, inflammation, and apoptosis markers in pancreatic islets	
		Increased expression level of insulin	

Wound healing	Multifloral honey, West Bengal (*Apis mellifera*)	Close resemblance of D-spacing and collagen diameter to normal skin collagen (scanning electron microscope observation)	[66]
	Multifloral honey, Iran (*Apis mellifera*)	Increased Oedema and necrosis	[67]
		Less infiltration of polymorphonuclear and mononuclear cells	
		Improve wound contraction	
		Increased epithelialisation	
		Increased concentrations of glycosaminoglycan and proteoglycan	
	Multifloral honey, Ibadan, Nigeria (*Apis mellifera*)	Increased granulation tissue in electroscalpel (ES) wound	[68]
		Increased fibroelastic tissue in honey treated wounds of ES group and honey treated wound cold scalpel	
	Tualang honey (*Apis dorsata*)	High tensile strength of colon anastomosis and fibroblast count	[69]
		High inflammatory cells	
	European bee honey (*Apis* spp.)	High hydroxyproline level in jaundiced animals treated with honey	[70]
		High bursting pressure	
	Stingless bee honey (*Trigona* spp.)	Prevent growth of rifampicin-resistant *S. aureus* and maintaining the sensitivity of *S. aureus* towards rifampicin	[71]
	Stingless bee honey (*Apis mellipodae*)	Showed effective effects in inhibiting growth of *S. aureus* and other pathogenic bacteria	[11]
	European bee honey (*Apis* spp.)	Stimulates healing process, clears infection, stimulates tissue regeneration, and reduces Inflammation	[58, 72, 73]

Anticancer	European bee honey (*Apis mellifera*)	Increased number of viable HepG2 cells in the human hepatoma cell (HepG2) treatment	[74]
		Improvement of the total antioxidant status	
		Caspase-3 activity is time and dose-dependent	
	Multifloral honey (*Apis mellifera*)	Increased rate of incidence, the efficacy to multiply, and the tumor size	[75]
	Stingless bee (*Trigona* spp.)	Reduced the total number of ACF and aberrant crypt and multiplicity of crypt	[76]
		No changes in the level of blood profile parameters, liver enzymes, and kidney functions	
	*Trigona incisa*, *Timia apicalis*, *Trigona fusco-balteata*, and *Trigona fuscibasis*	Increased cytotoxicity effects towards HepG2 cell line, while propolis crude extracts exhibit high cytoxicity effects towards all the human cancer cell lines	[77]
Treatment of eye diseases	Honeydew honey(*Apis mellifera*)	Bacterial flora in the conjunctival sac of patients with cataract and scheduled for vitrectomy was successfully eradicated after 7 days	[78]
	Australian and New Zealand honey (*Leptospermum* sp.)	Reduced formation of the whole colony-forming units in the eyelids and conjunctivae in patients with dry eye syndrome after one and three months of therapy	[79]
	Tualang honey (*Apis dorsata*)	No difference between the conventional treatment with Tualang honey eye treatment for chemical eye injury	[80]
	European bee honey (*Apis* spp.)	Corneas manifested an immediate regression of the corneal oedema	[81]
	Stingless bee (*Trigona* spp.)	Retardation of the cataract progress in 20% of the rats in the group that received honey for the opacification treatment	[82]
	Stingless bee honey (*Meliponula* spp.)	Reduced the infection time for eye diseases caused by *Staphylococcus aureus* and *Pseudomonas aeruginosa*	[83]

Fertility	Tualang honey (*Apis dorsata*)	Increased intromission and ejaculation percentage in rats	[84]
		Increased rate of fertility and mating	
	Honey Uremia, Iran (*Apis* spp.)	Reduced apoptosis and necrosis rate of the testicular cells in cells affected by noise stress and thereby increased cell viability	[85]
	Tualang honey (*Apis dorsata*)	Beneficial effects on level of corticosterone, pregnancy outcome, and adrenal histomorphometry	[86]
	Tualang honey (*Apis dorsata*)	Reduced cortisol and increased progesterone level of stress-induced female rats	[87]
		Increased testicular, epididymal weights, epididymal sperm count, motility, viability in nondiabetic, and sperm quality	[88]
